# Molecular Study of Selected Taxonomically Critical Taxa of the Genus *Iris* L. from the Broader Alpine-Dinaric Area

**DOI:** 10.3390/plants9091229

**Published:** 2020-09-18

**Authors:** Tim Weber, Jernej Jakše, Barbara Sladonja, Dario Hruševar, Nediljko Landeka, Slavko Brana, Borut Bohanec, Milenko Milović, Dalibor Vladović, Božena Mitić, Danijela Poljuha

**Affiliations:** 1 Institute of Agriculture and Tourism, Karla Huguesa 8, HR-52440 Poreč, Croatia; t.weber19@imperial.ac.uk (T.W.); barbara@iptpo.hr (B.S.); 2 Department of Life Sciences, Imperial College London, South Kensington Campus, London SW7 2AZ, UK; 3 Biotechnical Faculty, University of Ljubljana, Jamnikarjeva 101, SI-1000 Ljubljana, Slovenia; Jernej.Jakse@bf.uni-lj.si (J.J.); Borut.Bohanec@bf.uni-lj.si (B.B.); 4 Department of Biology, Faculty of Science, University of Zagreb, Rooseveltov trg 6, HR-10000 Zagreb, Croatia; dario.hrusevar@biol.pmf.hr (D.H.); bozena.mitic@biol.pmf.hr (B.M.); 5 Public Health Institute of the Istrian Region, Nazorova 23, HR-52100 Pula, Croatia; ddd@zzjziz.hr; 6 Public Institution Natura Histrica, Riva 8, HR-52100 Pula, Croatia; slavko.brana@natura-histrica.hr; 7 Medical and Chemical School, Ante Šupuk Street 29, HR-22000 Šibenik, Croatia; milenko.milovic@si.t-com.hr; 8 Nature History Museum Split, Poljana kneza Trpimira 3, HR-21000 Split, Croatia; dalibor@prirodoslovni.hr

**Keywords:** Iridaceae, Europe, chloroplast DNA, microsatellites, phylogeny, taxonomy

## Abstract

Some wild, morphologically diverse taxa of the genus *Iris* in the broad Alpine-Dinaric area have never been explored molecularly, and/or have ambiguous systematic status. The main aims of our research were to perform a molecular study of critical *Iris* taxa from that area (especially a narrow endemic accepted species *I. adriatica*, for which we also analysed genome size) and to explore the contribution of eight microsatellites and highly variable chloroplast DNA (*ndhJ*, *rpoC1*) markers to the understanding of the *Iris* taxa taxonomy and phylogeny. Both the microsatellite-based UPGMA and plastid markers-based maximum likelihood analysis discriminated three main clusters in the set of 32 analysed samples, which correspond well to the lower taxonomic categories of the genus, and support separate status of ambiguous regional taxa (e.g., *I. sibirica* subsp. *erirrhiza*, *I.* x *croatica* and *I.* x *rotschildii*). The first molecular data on *I. adriatica* revealed its genome size (2C = 12.639 ± 0.202 pg) and indicated the existence of ecotypes. For future molecular characterisation of the genus we recommend the utilisation of microsatellite markers supplemented with a combination of plastid markers.

## 1. Introduction

*Iris* L. (family Iridaceae) is a diverse genus with over 300 taxa distributed worldwide, mostly in the northern hemisphere [1,2]. In addition to conservational importance, many wild and cultivated taxa provide great horticultural value [3]. Phylogenetic and evolutionary studies of relationships of wild *Iris* taxa have long been challenging for several reasons. Namely, wide distribution, morpho-ecological diversity, multiple hybridisations, and convergent evolution processes, make definitive statements of the origin and evolution of taxa in the genus *Iris* very difficult [4,5]. To resolve a myriad of uncertainties and issues related to taxonomic and phylogenetic relationships within the genus *Iris*, extensive work was performed on morpho-anatomical features, palynology, phytochemical constituents’ analysis, cytogenetic traits, and molecular analysis [6,7,8,9]. Despite different approaches to lower (and individual) taxonomic categories, most authors agree on the classification of the genus *Iris* into six subgenera, which are divided into sections and series [1,10,11].

**Figure 1:**
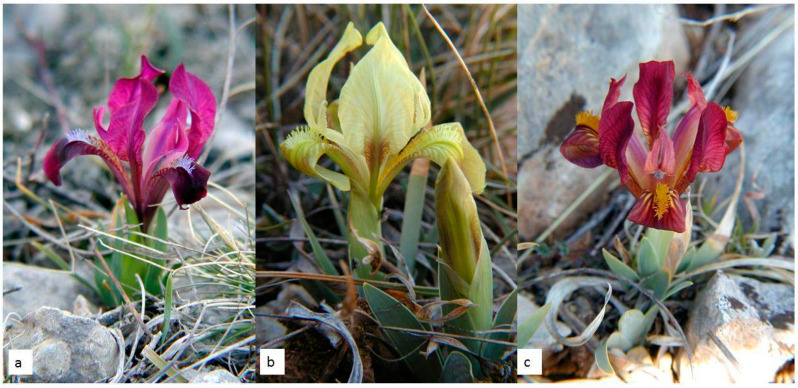
Narrow endemic wild Alpine-Dinaric endemic species *Iris adriatica*: (**a**–**c**) Individuals of different colours (Photo: Miroslav Mitić).

**Figure 2:**
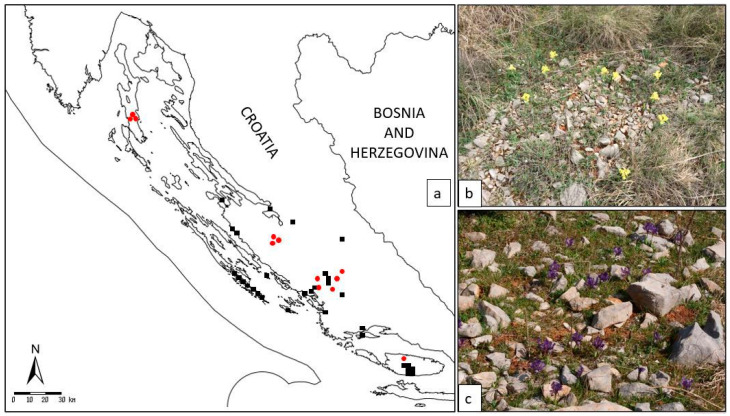
(**a**) Distribution map of the narrow endemic Alpine-Dinaric species *Iris adriatica* included in our study (all localities are in Croatia, and are incorporated in the national Flora Croatica Database (https://hirc.botanic.hr/fcd)—FCD; marks: 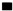
—earlier data from the FCD; 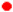
—localities of collected specimens in our study); (**b**) habitat on the locality Brnjica-Pokrovnik; (**c**) habitat on the island of Cres.

Most of the European native taxa of the genus *Iris* belong to the subgenus *Iris* L., section *Iris* L. (so-called “Pogoniris”), represented by numerous rhizomatous *Iris* taxa characterised by bearded outer tepals. Less prevalent on the European territory are taxa from the subgenus *Limniris* (Tausch) Spach, section *Limniris* (Tausch) Spach (so-called “Apogoniris”), which are rhizomatous irises whose outer tepals are without a beard [1,3]. The broad Alpine-Dinaric, as well as the surrounding Mediterranean and Pannonian area of Europe (where irises for our study were sampled) is characterised by peculiar eco-climate conditions which have caused a great morphological variability of some *Iris* populations and groups. Their variety has resulted in ambiguous systematic status of some regional, especially endemic, *Iris* taxa [5,8]. Some of them are recognised in the national and regional floras [12,13] and still have an unclear phylogenetic and classification status. Some of them neither are accepted in the World Checklist of Selected Plant Families [2], nor are molecularly researched in detail. Therefore we intended to molecularly study some, insufficiently researched and/or globally neglected taxa; namely: *I.* x *croatica* Horvat et M. D. Horvat (endemic in Croatia and Slovenia), *I. illyrica* Tomm. ex Vis.(endemic in Croatia, Slovenia, and Italy), *I. sibirica* L. subsp. *erirrhiza* (Posp.) Wraber (endemic in Bosnia and Herzegovina, Croatia, and Slovenia) and *I.* x *rotschildii* Degen (endemic in Croatia). However, in this study we paid special attention to the validly described [14] and accepted [2], molecularly unexplored endemic species *I. adriatica* Trinajstić ex Mitić (Figure 1 and Figure 2).

*I. adriatica* (Figure 1a–c) is a narrow endemic plant from the *I. pumila* complex, characterised by an extremely dwarf stem (one of the smallest species within the genus *Iris*) and relatively large yellow, violet, or purple solitary flowers (Figure 1a–c) [14].

It is confined to a few Croatian localities in the wider area of Dalmatia and classified as a NT (near threatened) species [13]. Given that the localities newly recorded by authors are spatially distant from the previously catalogued specimens (Figure 2), questions of subspeciation or higher-level genetic divergence can arise. All the more so as the recent metabolic profiling [15] revealed a notable diversity between the ecotypes and their pharmacological and chemotaxonomic potential.

Since the 1990s, when molecular biology techniques have become widely accessible, taxonomical biology has been driven towards using molecular methods to establish and re-establish evolutionary relationships between species [16,17]. Tang et al. [18] developed 400 ortholog-specific EST-SSR (Expressed Sequence Tag—Simple Sequence Repeats) markers, which can be reliably used to distinguish between the species in the *Iris* genus, providing a cheap and efficient way to resolve taxonomical discrepancies. Simple Sequence Repeats or microsatellites are present in most species; they are usually locus-specific, multiallelic, polymorphic, and co-dominant and are as such ideal candidates for discriminating between *Iris* species [19].

AChloroplast gene sequences are often used for plant phylogenetic studies and DNA barcoding because of the relatively low evolutionary mutation rates, their uniparental inheritance, high level of genetic diversity, and absence of recombination. Many candidate plastid regions have been suggested as the plant barcode and have as such been extensively tested [20,21,22]. However, to this end, a single marker has not yet been found which could reliably distinguish between a majority of plant species. Different combinatorial approaches have been used in different instances, to set on a final consortium [23]. Plastid DNA regions *rpoC1* and *ndhJ* used previously to evaluate plant phylogeny with low taxonomic variation [22] seemed appropriate for our study.

One of the basic genomic parameters that characterise the species and represent one of the important plant traits is the total amount of DNA in the unreplicated haploid or gametic cell nuclei, referred to as the C value or genome size [24]. Genome size data have numerous applications: They can be used in comparative studies on genome evolution, or as a tool to estimate the cost of whole-genome sequencing programs [25]. Currently, the largest updated plant genome size database—Plant DNA C-values database contains data for 12,273 species and among them 65 C-values for 44 species of genus *Iris* [26]. For most species involved in our study C-values are measured in several studies [27,28,29]. Different methods were used for the measurement of plant DNA content, but flow cytometry has become the method of choice due to its reliability, simplicity, and relatively low cost [30,31].

A noticeable lack of efforts to molecularly resolve remaining issues in *Iris* phylogeny and taxonomy on the Alpine-Dinaric area (including the adjacent areas of Mediterranean and the Pannonian Plain) in the context of conservation was extremely important when designing the study. Hence, to provide molecular insights into phylogenetic relationships of selected wild *Iris* taxa of the wider Alpine-Dinaric area, with a special emphasis on regional endemics and molecular evidence for their conservation, the aims of our research were: (i) To characterise representative and critical *Iris* taxa from the wider Alpine-Dinaric area by nuclear (SSR) markers; (ii) to clarify the genetic divergence within and between several wild (local endemic) and cultivated *Iris* populations through chloroplast DNA (cpDNA) markers; (iii) to present the first molecular description of a nearly threatened narrow endemic dwarf species *I. adriatica*; and (iv) contribute to the efforts of establishing optimal molecular markers for detecting taxonomic and phylogenetic relationships within critical taxa of the genus *Iris*.

## 2. Results

### 2.1. SSR Analysis

In total, 32 *Iris* samples across the Alpine-Dinaric region were analysed (Supplementary Table S1). Parameters of genetic diversity evaluation are presented in Table 1. SSR marker analysis was able to identify a total of 71 alleles (Supplementary Table S2). The observed number of alleles per locus ranged from 6 (at locus IM123) to 12 (at loci IM196 and IM327) with an average of 8.8 alleles and 4.3 effective alleles per locus. At locus IM348, out of eight alleles, allele 125 showed a frequency of 0.71; thus locus polymorphism information content (PIC) was 0.466, while at locus IM164, allele 324 showed a frequency of 0.68 resulting in locus polymorphism of PIC = 0.480. In general, the number of effective alleles was relatively low, indicating that rare and frequent alleles are present in the examined group of samples. The highest numbers of effective alleles (5.5 and 6.2) were observed at loci IM196 and IM327, respectively, where the frequencies of alleles were equally distributed. PIC values ranged from 0.466 (at locus IM348) to 0.845 (at locus IM391), indicating sufficient polymorphism information content of all loci. Loci IM164 and IM348 were moderately informative (0.25 < PIC < 0.5), while the rest were highly informative (PIC > 0.5). The expected heterozygosity varied between 0.490 (IM348) and 0.877 (IM391), with an average of 0.728. The highest observed heterozygosity (0.871) was found at locus IM123, and the lowest (0.129) was characteristic of locus IM164. The observed heterozygosity was lower than expected on all loci except IM123. The probability of identity (PI) values were in a range from 0.072 to 0.357, and the total PI calculated for all loci was 2.01 × 10^−7^, indicating a low probability of identical genotypes.

**Figure 3:**
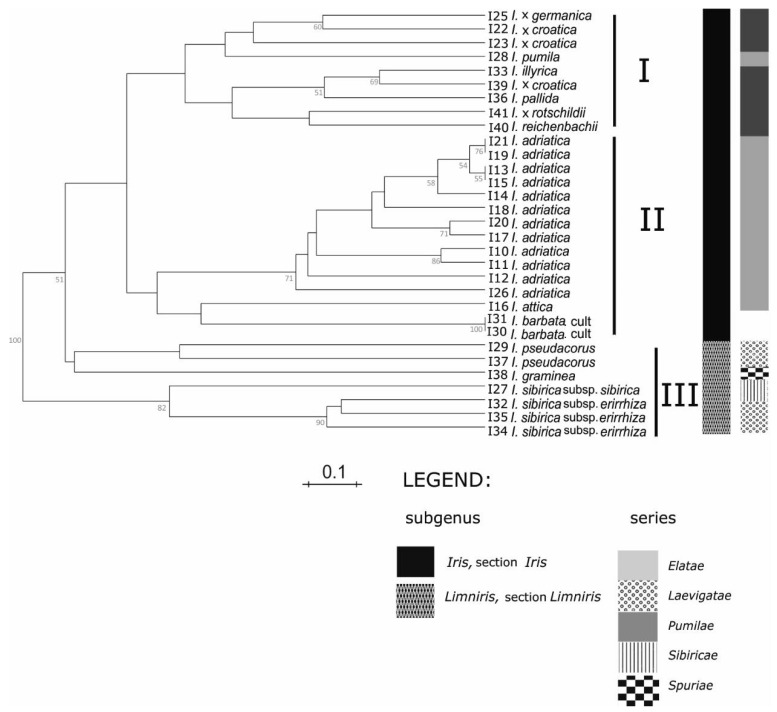
UPGMA dendrogram obtained with Dice’s similarity coefficient based on eight SSR markers for 31 out of 32 collected *Iris* samples (as explained in the Material and Methods section); Bootstrap percentages (>50) are shown in the nodes of the dendrogram; labels I–III denote major clusters.

The UPGMA clustering analysis (Figure 3) discriminated 28 genotypes and revealed three distinct groups of samples. The first cluster contained samples of mostly tall bearded Alpine-Dinaric taxa: *I.* x *croatica*, *I.* x *germanica* L., *I. illyrica*, *I. pallida* Lam., *I. pumila* L., *I. reichenbachii* Heuff., and *I.* x *rotschildii*, grouped in two subclusters. The second cluster (with several smaller subclusters) consists of all samples of narrow endemic dwarf species *I. adriatica*, its closely related species *I. attica* Boiss. & Heldr. as well as *I. barbata* cultivar, a horticulturally-widespread variety in the region. All samples of *I. sibirica* L. *sensu lato* grouped in the third cluster, together with *I. pseudacorus* L. and *I. graminea* L. within a separate subcluster.

### 2.2. Chloroplast Barcodes Analysis

**Figure 4:**
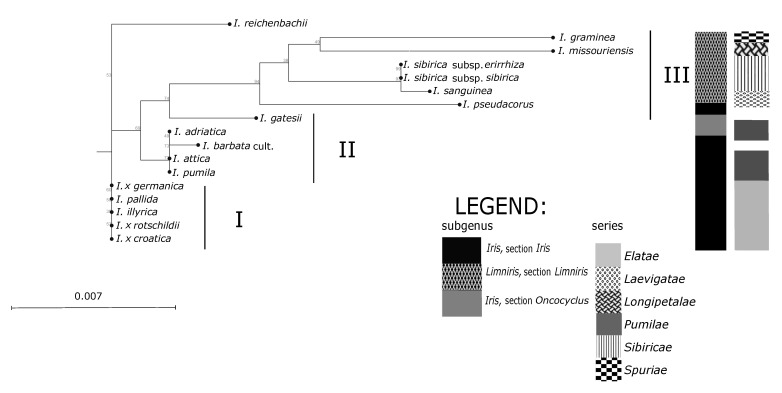
Maximum Likelihood (ML) tree of 32 *Iris* samples and sequences from NCBI (http://www.ncbi.nlm.nih.gov) (*I. missouriensis*, *I. sanguinea*, *I. gatesii*) based on two plastid markers (*rpoC1*, *ndhJ*). Bootstrap percentages are shown in the nodes of the dendrogram; labels I–III denote major clusters.

The maximum likelihood (ML) analysis was used in reconstructing phylogenetic relationships of a heterogeneous group of *Iris* species based on two plastid markers (*rpoC1*, *ndhJ*). ML analysis discriminated three major clusters of which seven groups of taxa and 10 different genotypes (Figure 4). In the ML dendrogram, three main groups of irises were discriminated, with *I. reichenbachii* separated from the rest. The first group consisted of five undiscriminated mostly Alpine-Dinaric species. Dwarf bearded irises *I. adriatica*, *I. pumila*, and *I. attica* were not separated and grouped with *I. barbata* cult. in the second cluster. Both subspecies of *I. sibirica* grouped in a third cluster, together with the out grouped *I. graminea* and *I. pseudacorus*. The samples accessed from NCBI gene repository *I. missouriensis* Nutt., *I. sanguinea* Hornem., and *I. gatesii* Foster were grouped appropriately, according to their classification within the genus *Iris*.

### 2.3. Genome Size

4′,6-diamidino-2-phenylindole (DAPI) fluorochrome is known to bind to DNA, specifically, to AT base pairs and therefore lower values for absolute genome size analysis are found [32]. The determination of total DNA content of plants from DAPI stained cells was: For *I. adriatica* 12.639 ± 0.202 pg (2C) and for *I.* x *germanica* 24.249 ± 0.163 pg (2C) respectively as compared to the *Pisum sativum* cv. Kleine Rheinländerin (9.07 pg/nucleus) internal standard.

## 3. Discussion

In our study, we applied 8 SSR markers developed by Tang et al. [18] which proved to be highly polymorphic and amplified alleles across the 39 *Iris* ecotypes and cultivars. We were not able to utilise the IM61 marker recommended but the remaining markers provided sufficient resolution to distinguish between our samples. We observed the greatest allelic diversity on IM196 and IM327 in concurrence with the aforementioned study; however, the observed number of alleles per locus in our study was significantly lower (average 8.8) suggesting greater phylogenetic similarity across all of our samples. Although it is comparable with the average number of alleles per locus observed within the group of 13 yellow-flag, Siberian, and tall-bearded *Iris* cultivars analysed by [18]. In our case, a small population size could be the reason for low allele frequency. Genetic similarity ranged from 0.23 to 0.8 and 0.26 to 1.00 among Alpine-Dinaric taxa from the subgenus *Iris* (section *Iris*) grouped in the UPGMA clusters I and II, respectively. The highest genetic similarity was intraspecific (Dice = 1; I19 and I21; I13 and I19; I30 and I31), whilst the lowest were interspecific (Dice = 0.23; I22 and I41 in cluster I; Dice = 0.26; I16 and I10; I16 and I11 in cluster II). Genetic similarity between endemic dwarf ecotypes of *I. adriatica* grouped within a separate subcluster, and correlated with the locations of origin, ranged from 0.55 to 1.00, implying significant and disperse genetic diversity among ecotypes. Taxa from the subgenus *Limniris* (section *Limniris*) displayed genetic similarity in a range from 0.07 to 0.72, the highest between samples of *I. sibirica* subsp. *erirrhiza*. Only a few SSR markers were needed to identify (distinguish) ecotypes and species.

The unique microsatellite profiles were established as described in the method section below, nevertheless, we acknowledge that the SSR analysis can differ from lab to lab as the method inherently produces high numbers of edge cases where a judgment call has to be made. An example of an edge case is the apparent presence of 3 alleles in what we presumed (and confirmed for *I. adriatica*) to be 2n = 2x species. As described, this was resolved by establishing a common SSR profile for those particular samples, since our subsequent analysis methods rely on the binary presence or absence of a particular allele and a presence of 3 alleles would likely confound the result and be factually incorrect. To resolve such an edge case a full sequencing run could reveal genomic mutations, such as translocation, or perhaps other properties of the genome at that position which would allow the probes to bind in this particular way. Further, as *I.* x *germanica* is a suspect tetraploid [5,18,33], the additional genetic information could skew the subsequent phylogenetic analysis as additional peaks appeared in positions only in one individual and could thus not be compared to any other values in the study, carrying an extremely low PIC. For our analysis such peaks were considered to be outliers; however, we are not suggesting they are not valid data in different subsamples.

This means that for any analysis the attribution of a particular profile needs to be internally consistent and cannot be used at face value form any further studies which want to include the same dataset. In our case, we employed the algorithm described in the methods to come to a conclusion which was cross-examined within the research group to preserve the established logic of sorting different cases. The final analysis of genetic relationship relies on the presence and absence of specific alleles so for our purposes the aim was to obtain the same profiles for the same species when attributing an SSR profile, without knowing which species the profile belongs to. Since a matching algorithm can only be established ad-hoc after accessing the reads, there is a potential to introduce some bias into edge-case decision making. Nevertheless, we are confident in our results several reasons; sample duplicates were included as an internal control and independently produced the same profiles using the same “blind” determination method, the chloroplast marker analysis largely produced the same clustering, profile differences between presumed same species are minimal, our described SSR relationship mirrors the relationships which were confirmed or predicted using taxonomic, botanical or other methods.

Different combination of chloroplast genome sequences were proposed for species discrimination, such as *rpoC1*, *rpoB*, and *matK*; *rpoC1*, *matK,* and *psbA-trnH;* [34] and *rbcL* and *trnH*-*psbA* [35]. In a recent review [23], authors Saddhe and Kumar discussed the utility of plastid markers to differentiate between different species within plant divisions, where they establish *ndhJ* as a good candidate marker for barcoding angiosperms. Additionally, *rpoC1* is often used as a supplementary marker to increase the barcoding depth of samples [36]. Plant Working Group (PWG) of the Consortium for the Barcoding of Life (CBOL) recommended the combination of *rbcL* and *matK* as the plant barcode [20], while *rpoB* and here applied *rpoC1* showed markedly lower discriminatory power. Chloroplast marker *matK* is recommended as one of the best DNA barcoding candidates for species discrimination [20,37]. However, this chloroplast region proved to be difficult to amplify and sequence in certain taxa, and additional universal primers and optimisation of PCR reactions were necessary [38,39]. In our study, the preliminary amplification of *matK* sequences was unsuccessful and the testing of additional plastid markers is foreseen. However, the combination of *ndhJ* and *rpoC1* revealed to be adequate for discrimination up to the series taxonomic level, indicating the possibility of applying additional candidates for the species discrimination. As discussed, a plastid marker with sufficient resolution would be operationally favourable for widespread utility in discriminating between different species. Up to date a few phylogenetic studies based on chloroplast markers were carried out on *Iris* [6,40,41,42]. Neither *ndhJ* nor *rpoC1* was not tested in any *Iris* genus study.

Groupings of the *Iris* taxa from the broader Alpine-Dinaric area, observed in our research by both sets of molecular markers (Figure 3 and Figure 4), mostly correspond to proposed phylogenetic relationships based on palynological features [8]; a clear distinction between the subgenera *Limniris* and *Iris* and within the majority of the lower taxonomic *Iris* categories of sections and series emerges. The anticipated exception is the position of analysed NCBI sequence of Middle Eastern species *I. gatesii* (Figure 4), which separated within the subgenus *Iris* in an individual cluster, as it belongs to the different series *Oncocyclus* (Siemssen) Baker [1]. However, the unexpected exceptions are positions of the species *I. pumila* based on SSR markers (Figure 3), and of *I. reichenbachii* based on ML analysis (Figure 4). Molecular analysis of both sets of markers (Figure 3 and Figure 4) in principle resulted in the creation of three main clusters: Two of three clusters covering rhizomatous taxa from the subgenus *Iris*, section *Iris*, with a beard (“Pogoniris”, [3]), while the taxa from the subgenus *Limniris*, section *Limniris*, rhizomatous irises with falls without a beard (“Apogoniris”, [3]) were grouped in the third cluster (Figure 3 and Figure 4). For the ML analysis control NCBI sequences: Of *I. sanguinea* (subgenus *Limniris*; sect. *Limniris*, series *Sibiricae* (Diels) Lawrence) and *I. missouriensis* (subgenus *Limniris*; sect. *Limniris*, series *Longipetalae* (Diels) Lawrence), grouped with other members of the same subgenus (Figure 4); and of *I. gatesii* (subgenus *Iris*; section *Oncocyclus*) made a separate branch between samples of “Apogoniris” and the rest of the “Pogoniris” (Figure 4). Such results are in agreement with previous studies and monographs of the genus *Iris* [1,3,11,41,43].

Within the subgenus *Iris*, section *Iris*, on the series level, one cluster (based on both sets of molecular markers; Figure 3 and Figure 4) comprises the group of mostly tall bearded irises and covers the series *Elatae* Lawr. [10]. The second cluster covers the group of dwarf bearded irises and matches the series *Pumilae* Lawr. [10], except for *I. pumila* grouping in the first cluster based on SSR markers analysis (Figure 3). However, plastid markers (Figure 4) did not discriminate analysed taxa within neither series *Elatae* (the only exception is *I. reichenbachii*) nor *Pumilae*. In our study chloroplast markers *ndhJ* and *rpoC1* provide a weaker resolution into the species, concurrent with other authors [22]; however, we acknowledge that the analysis of sequence data is quicker and much less prone to human error and enables clustering comparison across different studies if the sequences are made publicly available. Further, our study looked at only two plastid regions, as compared to eight microsatellite loci. Therefore, we would recommend the utilisation of SSR markers for subsequent analysis supplemented by a plastid marker combination for the genus *Iris*, until a single plastid marker combination is established as a convention.

According to SSR markers analysis (Figure 3), within the cluster I, two subgroups were formed: In the first are two samples of tall bearded *I.* x *croatica*, *I.* x *germanica,* and, unexpectedly, dwarf bearded *I. pumila*, whereas one sample of *I.* x *croatica* is grouped with other analysed tall bearded irises within the second subgroup. Although its taxonomic position is critical and still unresolved, the taxon *I.* x *croatica* is considered as a native endemic taxon in northern Croatia and Slovenia [12,13,44]. Likely due to morphological similarities, it is often mixed with and named as a synonym for *I.* x *germanica* [1,2,5,13], which is, in our opinion, distributed worldwide only as a cultivated hybrid species [1,9]. The fact that the WCSP [2] wrongly “declares” *I. croatica* Horvat & M.D. Horvat as an illegitimate name, due to an incorrect replacement with *I. croatica* Prodan, provokes further taxonomic confusion [45], explained in detail in [5]. The close relationship between *I*. x *croatica* and *I.* x *germanica* was noticed by examining both plant and pollen morphology [8] (B. Mitić, personal observations) and is confirmed with our results—their joint sub clustering (Figure 3). However, they are both tetraploids of yet unresolved origin with reported chromosome numbers of 2n = 44 for *I.* x *germanica,* and 2n = 48 for *I.* x *croatica* [5,46]. Two earlier speculations about (auto) tetraploid origin of *I.* x *croatica* both agreed that the progenitor species for that hybrid is *I. pallida*, although this is yet to be cytogenetically confirmed [5,8]. Grouping a sample of *I.* x *croatica* together with *I. pallida* and *I. illyrica* within the second subgroup in our results (Figure 3) confirms the proximity of tetraploid *I.* x *croatica* and presumed progenitor species *I. pallida*.

Considering the clear discrimination within lower taxonomic subgroups such as series, obtained by the applied marker systems (Figure 3), the status of other closely related taxa from the so-called *I. pallida* complex could be discussed. Taxonomic relationships within the complex have not been fully explored and it is not yet clear whether the taxa of this complex have the status of species or subspecies. Namely, the majority of taxa from this complex (including representatives from our research—*I. pallida* and *I. illyrica*) were defined at the species level and extracted, apart from the series *Elatae* into the new series *Pallidae* (A. Kern.) Trinajstić [47]. Although earlier taxonomic researches of *I. pallida* complex [48,49] have supported such taxonomic treatment of its taxa, a later palynological study [8] indicated their return into the status of subspecies level (as classified by WCSP [2]), and of the series *Pallidae* back into the series *Elatae*. Results of our study are in accordance with the last hypothesis as both marker systems (Figure 3 and Figure 4) grouped members of those series closely together.

The taxon *I.* x *rotschildii* from the series *Elatae* also garners considerable attention in the context of this study. So far, this narrow endemic iris is known from a single locality on Mt. Velebit (Croatia). It is described as a natural hybrid between species *I. illyrica* and *I. variegata* L. [1,50] with observed morphological, palynological, and cytogenetic variabilities [46]. Some of the mentioned features confirm the hybridogenous origin of this taxon. Despite this, no further molecular studies have been done on the taxon to confirm its claimed status. This is the likely reason it was recently considered as a synonym of *I.* x *germanica* by WCSP [2]. Unfortunately, due to hard-to-reach mountainous terrain (with mines still present in the area) and the small number of specimens in the only known population on Mt. Velebit (B. Mitić, personal observations), only one sample of this taxon was included in our analysis. Bearing this in mind, the SSR profile of *I.* x *rotschildii* that shares at least one allele on all analysed loci with *I. illyrica* as well as their position in the same UPGMA subcluster additionally support their parent-sibling relationship (Figure 3). Moreover, although *I.* x *germanica* and *I.* x *rotschildii* are presumed synonyms [2], their discrimination by SSR could disprove that assumption and would favour the placement of *I.* x *rotschildii* within a separate taxonomic position. However, further extensive detailed molecular study of *I.* x *rotschildii* and its presumed parents is needed to confirm both its separate taxonomic status and its difference with *I.* x *germanica*.

Furthermore, unexpected discrepancies occur in the placement of *I. reichenbachii*, which was positioned in the same UPGMA subcluster as *I. illyrica*, *I. pallida*, and *I.* x *rotschildii* (Figure 3) and also as an outgroup in ML dendrogram (Figure 4). Namely, *I. reichenbachii* is native in mountainous regions of the Balkan Peninsula and SW Romania, known as parental species of some natural hybrids [5], and according to [10] was firstly placed in the series *Pumilae.* However, according to both chromosome numbers 2n = 24, 48 [43] and pollen analyses [8] it seemed to fit better in the series *Elatae*. Nevertheless, outgrouping of *I. reichenbachii* in our ML analysis (Figure 4) might indicate its specific position between two series that still needs to be explored, as it has the same number of chromosomes [5] and pollen grains [8] as tall bearded irises and is morphologically quite dwarfish [43]. Further, its genome size (1C value) is intermediate between some members of both series *Elatae* and *Pumilae* [33].

Cluster II in our study (Figure 3 and Figure 4) covers mostly dwarf bearded irises. However, except for *I. pumila* based on SSR markers, grouping within the first cluster (Figure 3), together with tall bearded *I.* x *croatica* and *I.* x *germanica*. Given current evidence, we speculate that the grouping may have happened due to the normalisation of the chromosomal content applied, and treatment of SSR data as codominant, with maximally two alleles counted. An additional element could be genetic variability of *I. pumila*, evident from genome size of this tetraploid species (2n = 32), differing in several previous studies (e.g., 1C = 13.20 pg [27]; 1C = 6.81 pg [33]; 1C = 10.64 pg [51]). Furthermore, this taxon is supposed to have the same hypothetical ancestor as tall bearded irises (with x = 4 [3,43]), and is often known as the parental species (together with some tall bearded irises as second parents) of many native and artificial hybrids [43].

On the contrary, all other investigated samples of dwarf bearded irises of the series *Pumilae* [10] grouped in a separate cluster II based on plastid markers (Figure 4). Such results are in accordance with pollen morphology of dwarf bearded irises [8,52] and confirm their separate taxonomic position, and belonging to the same *I. pumila* complex [14]. Since *I. attica* is the only member of the complex with the status of a subspecies, and with others having equal rank of species in the WCSP [2], our results (Figure 3 and Figure 4) suggest that they should be treated at the same taxonomic rank. Therefore, further research is needed to corroborate (or disprove) our statement about taxonomic relationships within the whole *I. pumila* complex.

Meanwhile, special attention in our study was dedicated to one member of the complex—a relatively-recently described diploid (2n = 16) species *I. adriatica* [14], native and endemic to Croatia. Namely, to prepare the basis for its conservation, because of its nearly threatened species status [13], we were particularly focused on its molecular features. Evidence about taxonomic and phylogenetic values of palynological and phytochemical features of *I. adriatica* are well documented [8,15]. However, thus far, this species has not been researched on a molecular level. In the present results (Figure 3) we documented diversity of different populations of the species *I. adriatica*, showing the existence of geographical ecotypes. In particular, the UPGMA grouping (Figure 3) of established ecotypes corresponds well with the geographical origins of the samples (Figure 2, Supplementary Table S1): The island populations (sample numbers I26 island of Brač; I10, I11, and I12 island of Cres) have separated from the land coastal populations (Figure 3, Supplementary Table S1, other samples). Therefore, we assume that island populations might be a specific ecotype of the typical species. Within inland populations, we were particularly interested in the population of the hinterland population “Brnjica-Pokrovnik”, which has been singled out as an ecotype based on phytochemical analysis [15]. In our analysis (Figure 3, sample no. I18) it has a separate branch in the dendrogram, although it is “surrounded” by other inland populations. Therefore, it is obvious that potential inland ecotype(s) require additional investigations. One more reason in favour of the separation of ecotypes is the fact that “Brnjica-Pokrovnik” population is growing on an open calcareous meadows (mainly belonging to the *Festuco-Koelerietum splendentis* Horvatić 1963 association), whilst the rest of the researched populations grow on limited rocky pastures and hills (mainly belonging to the *Stipo-Salvietum officinalis* Horvatić 1985 association), very often endangered by the succession, i.e., overgrowth with macchia.

Furthermore, in this study we present the first genome size estimation of *I. adriatica* measured by flow cytometry and expressed according to [24] as 2C value = 12.639 ± 0.202 pg. Observed value of genome size for *I. adriatica* we could hardly compare with values of all other members of the complex *I. pumila*, since the data are known only for tetraploid species *I. pumila* [27,33,51]. However, as previously mentioned, data for this tetraploid species indicates its variability. Our results of genome size value for diploid species *I. adriatica* are the first data about genome size for this strictly endemic, near threatened species and should contribute to its future conservation. The 1C value of *I. adriatica* is similar to that of tetraploid *I. pumila* obtained by [33]. Such results should confirm belonging of both species to the same complex. Additionally, similar deviations in 1C values as in the species *I. pumila*, were observed for the species *I*. x *germanica*: Our results of 2C = 24.249 pg for this “control” species could be compared to the result (1C = 12.45) of [27], while the value of 1C = 5.87 for the same species was observed by [33]. Therefore, the genome sizes of critical taxa of the genus *Iris* require further, more complex research.

In our results within the third cluster (Figure 3 and Figure 4) all samples of so-called “Apogoniris” taxa [3] grouped together, further all are representatives of the subgenus *Limniris*, section *Limniris*. Such results are in accordance with some previous research of molecular phylogeny of these taxa [40,53,54]. Additionally, our analysis based on both sets of markers (Figure 3 and Figure 4) resulted with different subclusters within the subgenus *Limniris*. Namely, mentioned subgroups correspond well to the series as a lower taxonomic level (according to [1,10]): *Laevigatae* (Diels) Lawrence (*I. pseudacorus*), *Sibiricae* (Diels) Lawrence (both subspecies of *I. sibirica*), and *Spuriae* (Diels) Lawrence (*I. graminea*). The analysed NCBI sequences of “Apogoniris” taxa (*I. missouriensis* and *I. sanguinea*) additionally support that distinction (Figure 4), they grouped with other members of the subgenus *Limniris*, section *Limniris*. Moreover, *I. sanguinea*, which belongs to the series *Sibiricae* [1], grouped close to other members of this series.

Furthermore, all samples of *I. sibirica sensu lato* (series *Sibiricae*) grouped apart from other members of the subgenus *Limniris* (Figure 3 and Figure 4), and created further subclusters (Figure 3). This was especially interesting because of the still unclear position of the Alpine-Dinaric mountain populations described as subspecies of the typical *I. sibirica* species [55]. Although plastid markers (Figure 4) did not discriminate *I. sibirica* subspecies, the results of SSR analysis (Figure 3) confirmed their differentiation. This is also in accordance with the presumption that *I. sibirica* subsp. *erirrhiza* might be a mountain ecotype [46], which differs from the typical lowland subspecies *I. sibirica* subsp. *sibirica* [55]. This is particularly interesting for further conservation of wild, especially endemic irises from that area. Namely, *I. sibirica* subsp. *erirrhiza* was found only in several localities in Bosnia and Herzegovina, Croatia, and Slovenia where it might be an endemic taxon [46,55]. The subclustering of *I. sibirica* subsp. *erirrhiza* samples in our research and an extra subcluster of typical *I. sibirica* subsp. *sibirica* (Figure 3) additionally confirms this distinction of subspecies as ecotypes. Unfortunately, in our study we did not have a sample of the population of *I. sibirica* subsp. *erirrhiza* from Mt. Bjelolasica (Croatia), the supposed link between the subgenera *Limniris* and *Iris* in the territory of Southern Europe [8]. Further research focused on broader ecotype samples of *I. sibirica sensu lato* is needed to give a better insight into the phylogenetic structure within this complex taxon.

Regarding other representatives of the subgenus *Limniris* in our study, we can comment on the specific position of the species *I. graminea*, which separated in the distinct cluster in both trees (Figure 3 and Figure 4). Therefore, our results might support the hypothesis that the species *I. graminea* is probably the most primitive member of the subgenus *Limniris* on the Southern European territory [8]. Besides this, our analysis of microsatellites (Figure 3) might also confirm the opinion based on palynological observations, that the subgenus *Iris* is more advanced than the subgenus *Limniris* [8,56].

In closure, we can confirm that our results of the molecular study of Alpine-Dinaric taxa of the genus *Iris* correspond well with their positions within the subgenera *Iris* and *Limniris*, and are in accordance with some other recent molecular researches of taxa of the genus *Iris* [41,57]. Additionally, our results present the first molecular data on narrow endemic and near threatened species *I. adriatica* and also support the separate taxonomic status of investigated ambiguous regional taxa (e.g., *I. sibirica* subsp. *erirrhiza*, *I.* x *croatica* and *I.* x *rotschildii*).

## 4. Materials and Methods

### 4.1. Plant Material and DNA Extraction

Plants of the genus *Iris* distributed across the broader Alpine-Dinaric region were collected either in their natural habitats during the vegetation seasons 2016–2018, retrieved from botanical collections of the National Botanical gardens in Zagreb (Croatia) and Ljubljana (Slovenia) (Supplementary Table S2). Most vouchers are live specimens deposited within the *Iris* collections of the mentioned Botanical Gardens in Zagreb and Ljubljana, and one in the private garden of the corresponding author. Herbarium voucher specimens are deposited in the herbarium of the Istrian Botanical Society, Vodnjan, Croatia (not yet registered in the Index Herbariorum). Total genomic DNA was isolated from 25–100 mg dried or fresh leaves, depending on the sample, using the commercial kit PureLink^®^ Plant Total DNA Purification Kit (Invitrogen^TM^; Waltham, Massachusetts, USA), in accordance with the manufacturer’s instructions. One sample (I24; *I. sibirica* subsp. *sibirica*; Supplementary Table S2) was excluded from SSR analysis due to poor imaging signals.

### 4.2. Microsatellite and Chloroplast Barcodes Amplification

Eight SSR markers [18] were used for genotyping (Supplementary Table S2), following the optimised procedures described in [18]. Forward SSR primers were end-labelled with one of three fluorophores, 6FAM, HEX, or TAMRA (Supplementary Table S3). Briefly, the initial denaturation step was performed at 95 °C for 3 min, followed by 1 cycle of 94 °C for 30 s, 55–64 °C (depending on optimal annealing temperature (T_a_)) for 30 s and 72 °C for 45 s. The annealing temperature was decreased 1 °C per cycle in subsequent 7 cycles until reaching the optimal T_a_ (Supplementary Table S3) at which 35 cycles were carried out, with a final extension at 72 °C for 20 min. The PCR products were checked on 2% agarose gels to confirm amplification. The length of the PCR products was determined through capillary gel-electrophoresis (Macrogen Europe B.V., Amsterdam, the Netherlands). SSR alleles were resolved on the ABI3730XL DNA Analyser (Applied Biosystems^TM^; Waltham, Massachusetts, USA), using GeneMarker^®^ Software V2.7.0 (SoftGenetics, State College, Pennsylvania, USA) and 400HD ROXTM dye-labelled internal size standard marker. SSR peak estimates were determined using inbuilt software on pre-set settings. Each peak was individually evaluated. False positives were eliminated by looking at peak values appearing at the same position in reads where no SSR probe was present for a particular analyte, judged to be innate background. Due to slight shifts occurring at each read, peaks from different runs, which were consistently different in length were judged to be the same SSR profile [58]. All samples were described using a maximal value of two alleles at each SSR locus examined normalised to a 2n = 2 × chromosomal content (Supplementary Table S2). Where more than two alleles (peaks) were apparent their pattern was cross-examined with other available samples to determine their unique descriptive allelic values.

A combinatorial approach of *ndhJ* and *rpoC1* plastid markers (Supplementary Table S3) was used for barcoding according to the procedure of [59]. The procedure consisted of an initial denaturation step at 94 °C for 5 min, followed by 35 cycles of 94 °C for 30 s, 55 °C for 30 s, 72 °C for 60 s and a final extension step at 72 °C for 10 min. Before sequencing PCR products were additionally purified using exonuclease I and shrimp alkaline phosphatase to remove unincorporated nucleotides and primers. The barcodes were Sanger Sequenced using ABI 3130XL capillary machine (Biotechnology Faculty, University of Ljubljana, Ljubljana, Slovenia) and submitted to GenBank (Supplementary Table S2). Further three additional sequences (*I. gatesii*, GenBank: KM014691.1; *I. missouriensis*, NCBI Reference Sequence: NC_042827.1; *I. sanguinea*, NCBI Reference Sequence: NC_029227.1) were mined from the NCBI repository. Sequences were aligned using Codon Code Aligner V9.0.1 (CodonCode Corporation, Centerville, MA, USA).

### 4.3. Data Analysis

Genetic parameters were calculated for 32 *Iris* samples over eight microsatellite loci (Table 1). The number of amplified microsatellite alleles (n), number of effective alleles (n_e_), observed heterozygosity (H_o_), and expected heterozygosity (H_e_) were calculated using POPGENE, version 1.32 [60]. Polymorphic Information Content (PIC) was calculated with the program Cervus, Version 3.0.7 [61] and probability of identity (PI) was determined using IDENTITY v.1.0 program [62]. Genetic distances between all pairwise combinations of the samples were calculated using Dice’s coefficient of similarity. The dendrogram was constructed from the resultant matrices via the UPGMA distance-matrix method using the PAST software [63]. Statistical support for the tree topology was assessed by 1000 bootstrap replicates. The two chloroplast loci (*rpoC1* and *ndhJ*) sequence data were aligned using the “Create Alignment” algorithm implemented in CLC Genomics Workbench 20.0.2. Alignments were joined together and a Maximum Likelihood Neighbour-Joining tree was constructed using the “Maximum Likelihood Phylogeny” algorithm of CLC using the Jukes–Cantor nucleotide substitution model.

### 4.4. Genome Size Analysis

The DNA content of *I. adriatica* and *I.* x *germanica* plants were analysed by flow cytometry analysis according to the method reported in [32]. A portion of the fresh young leaves tissue of approximately 1 cm^2^ was used in sample preparation. For an internal standard, the *Pisum sativum* cv. Kleine Rheinländerin (9.07 pg/nucleus) was used for reference. Both the sample and the standard were chopped finely using a razor and released into 0.1 M citric acid containing 0.5% Tween 20. The homogeneous mixture was filtered through a 30-μm nylon filter removing larger particles. 4′,6-diamidino-2-phenylindole (DAPI) was used as the genome staining dye. A 3–4-fold volume of staining buffer containing 4 μg ml^−1^ of DAPI in 0.4 M Na_2_HPO_4_ × 12H_2_O was added to the specimens.

Samples were analysed with a Partec CyFlow^®^ Space flow cytometer using linear scale. FloMax^®^ software (Partec, Münster, Germany) was used for the calculation of relative nuclear DNA content.

## 5. Conclusions

In the present molecular study of selected representative and critical *Iris* taxa from the wider Alpine-Dinaric area, we enhanced the current knowledge and understanding of the genus *Iris* taxonomy and phylogeny of the area; important for their further protection and conservation in the study area. Our research showed taxonomic positions of investigated taxa within the genus *Iris*, which is mostly in accordance with previous comprehension of the genus *Iris*. We were especially focused on getting the first molecular data on the nearly threatened narrow endemic dwarf species *I. adriatica*, hitherto molecularly unexplored. The results of molecular analysis showed that the 2C value for this species is 12.639 ± 0.202 pg, pointing to its relationship with other dwarf irises from the *I. pumila* complex, and indicating the existence of ecotypes. Additionally, we stressed some, presently unresolved, key taxonomic questions about certain critical groups and/or taxa of the genus *Iris* from that area, and the most pertinent are: Taxonomic and phylogenetic relationships of some complex *Iris* groups from this area (e.g., I. x *germanica*, *I. pallida*, *I. pumila* and *I. sibirica* groups) and the taxonomic status of regionally recognised, but globally neglected endemic taxa: *I. sibirica* subsp. *erirrhiza*, and natural hybrids *I.* x *croatica* and *I.* x *rotschildii*. For mentioned groups and taxa our study establishes baseline taxonomic and phylogenetic relationships across the Alpine-Dinaric region, but more precise confirmation of their phylogenetic and taxonomic status require further, more complex molecular analysis on a broader set of *Iris* samples. Regarding the contribution to the efforts of establishing optimal molecular markers for detecting taxonomic and phylogenetic relationships within critical taxa of the genus *Iris*, we would recommend the utilisation of SSR markers for subsequent analysis supplemented with a combination of plastid markers until a plastid marker combination for the genus is established and fully validated as convention. Chloroplast markers *ndhJ* and *rpoC1* provide a weaker resolution into the species; however, analysis of sequence data is quicker and much less prone to human error. Further, our SSR study looked at 8 microsatellite loci as compared to two plastid regions. Chloroplast markers can give further context to SSR analysis and provide independent control despite their lower resolution as they can confirm broader clusters. For future studies of the genus *Iris* we would additionally recommend the inclusion of other appropriate barcoding regions to serve the same purpose and hopefully increase the sequencing resolution.

Molecular evidences obtained in this study, besides contribution to the knowledge on taxonomy and phylogeny of the genus *Iris* in the Alpine-Dinaric, Mediterranean and Pannonian area, should also help in further understanding about the importance of wild, especially endemic *Iris* taxa and encourage their more intensive conservation efforts.

## Figures and Tables

**Table 1:** Values of observed (H_o_) and expected (H_e_) heterozygosity, number of alleles (n), effective number of alleles (n_e_), polymorphic information content (PIC), and probability of identity (PI) of 8 microsatellite loci for all studied samples of the Alpine-Dinaric taxa of the genus *Iris*.

Locus	n	n_e_	H_o_	H_e_	PIC	PI
IM93	9	4.1	0.452	0.769	0.727	0.131
IM123	6	4.0	0.871	0.763	0.712	0.172
IM164	7	2.0	0.129	0.518	0.480	0.292
IM196	12	5.5	0.500	0.833	0.805	0.079
IM200	8	3.4	0.387	0.721	0.672	0.178
IM327	12	6.2	0.593	0.855	0.821	0.085
IM348	8	1.9	0.194	0.490	0.466	0.357
IM391	9	7.2	0.714	0.877	0.845	0.072
**Average**	8.8	4.3	0.480	0.728	0.691	-
**Total**	-	-	-	-	-	2.01 × 10^−7^

## Supplementary Materials

The following are available online at https://www.mdpi.com/2223-7747/9/9/1229/s1, Table S1: Alpine-Dinaric taxa of the Genus *Iris* used in the present molecular study; Table S2: Genotypes of the analysed *Iris* samples at eight microsatellite loci (allele sizes in bp); Table S3: SSR and chloroplast markers used in the present molecular study of the Alpine-Dinaric taxa of the genus *Iris.*
