# Supplementary material for: Molecular Study of Selected Taxonomically Critical Taxa of the Genus Iris L. from the Broader Alpine-Dinaric Area
Source: Plants (Basel). 2020 Sep 18;9(9):1229. doi: 10.3390/plants9091229 (PMC7570032; doi:10.3390/plants9091229)
Supplement: Supplementary file 1 [file plants-09-01229-s001.zip › SUPPLEMENTARY FILES corr/Table S1_corr.docx]

**Table S1.** Alpine-Dinaric taxa of the Genus *Iris* used in the present molecular study, including information about the origin of studied material or collection where it is maintained  (with coordinates), voucher information, as well as GenBank accession numbers (N - Latitude; E - Longitude; BGLjU – Botanical Garden Ljubljana; BGZG – Botanical Garden Zagreb; l.c. – *locus classicus*; s.n. - *sine numero*).

| **DNA label** | **Taxon** | **Origin (Collection)** | **Coordinates N** | **Coordinates E** | **Voucher**  **No.** | **GeneBank**  **accession No** | |
| --- | --- | --- | --- | --- | --- | --- | --- |
|  |  |  |  |  |  | ***rpoC1*** | ***ndhJ*** |
| I10 | *I. adriatica* Trinajstić ex Mitić | Croatia, island of Cres | 44.751.393 | 14.419.169 | Istrian Botanical Society, Vodnjan, Herbarium, 2667 | MT732229 | MT732242 |
| I11 | *I. adriatica*  Trinajstić ex Mitić | Croatia, island of Cres | 44.752.224 | 14.425.001 | Istrian Botanical Society, Vodnjan, Herbarium, 2668 | MT732227 | MT732236 |
| I12 | *I. adriatica*  Trinajstić ex Mitić | Croatia, island of Cres | 44.747.780 | 14.422.778 | Istrian Botanical Society, Vodnjan, Herbarium, 2671 | MT732226 | MT732238 |
| I26 | *I. adriatica*  Trinajstić ex Mitić | Croatia, island of Brač | 43.358.429 | 16.597.101 | 2016/10 | MT732217 | MT712014 |
| I13 | *I. adriatica*  Trinajstić ex Mitić | Croatia, Nadin | 44.021.138 | 15.573.188 | Istrian Botanical Society, Vodnjan, Herbarium, s.n. | MT732228 | MT732230 |
| I14 | *I. adriatica*  Trinajstić ex Mitić | Croatia, Nadin | 44.021.138 | 15.573.188 | Istrian Botanical Society, Vodnjan, Herbarium, s.n. | MT732225 | MT732235 |
| I15 | *I. adriatica*  Trinajstić ex Mitić | Croatia, Nadin | 44.021.138 | 15.573.188 | Istrian Botanical Society, Vodnjan, Herbarium, s.n. | MT732224 | MT732243 |
| I19 | *I. adriatica*  Trinajstić ex Mitić | Croatia, near Šibenik, Trtar Mt. | 43.733.216 | 15.999.940 | 2016/8 (BGZG) | MT732222 | MT732237 |
| I17 | *I. adriatica*  Trinajstić ex Mitić | Croatia,  Brnjica | 43.786.332 | 16.028.191 | 2016/6 (BGZG) | MT732221 | MT732240 |
| I18 | *I. adriatica*  Trinajstić ex Mitić | Croatia, Brnjica Pokrovnik | 43.812.524 | 16.053.133 | 2016/7 (BGZG) | MT732220 | MT732231 |
| I20 | *I. adriatica*  Trinajstić ex Mitić | Croatia, Šibenik (Njivice, mons Smričnjak; l.c.) | 43.749.710 | 15.881.098 | 2016/2;14341 (BGZG) | MT732219 | MT732233 |
| I21 | *I. adriatica*  Trinajstić ex Mitić | Croatia, Bilice | 43.789.680 | 15.892.601 | 2016/3 (BGZG) | MT732218 | MT712013 |
| I16 | *I. attica* Boiss. & Heldr. | Greece, Peloppones, Sparta | 37.042.399 | 22.254.699 | 2008/2 (BGZG) | MT732223 | MT732241 |
| I30 | *I. barbata* cult. | Croatia, Istria, Poreč | 45.227.254 | 13.614.557 | s.n. (Croatia, Poreč, private garden) | MT732212 | MT732239 |
| I31 | *I. barbata* cult. | Croatia, Istria, Poreč | 45.227.254 | 13.614.557 | s.n. (Croatia, Poreč, private garden) | MT732213 | MT732234 |
| I22 | *I.* x *croatica* Horvat et M. D. Horvat | Croatia, Bot. Garden Zagreb | 45.804.818 | 15.973.182 | 92/101 (BGZG) | MT732207 | MT732255 |
| I23 | *I.* x *croatica*  Horvat et M. D. Horvat | Croatia, Strahinjščica Mt. | 46.114.952 | 15.543.111 | 92/103 (BGZG) | MT732208 | MT732256 |
| I39 | *I.* x *croatica*  Horvat et M. D. Horvat | Slovenia, Bot. Garden Ljubljana | 46.040.425 | 14.514.397 | s.n. (BGLjU) | MT732210 | MT732254 |
| I25 | *I.* x *germanica* L. | Croatia, Bot. Garden Zagreb | 45.804.818 | 15.973.182 | 92/105 (BGZG) | MT732214 | MT732252 |
| I38 | *I. graminea* L. | Slovenia, Nanos Mt., Bot. Garden Ljubljana | 46.040.425 | 14.514.397 | s.n. (BGLjU) | MT732198 | MT712012 |
| I33 | *I. illyrica* Tomm. ex Vis. | Slovenia, Bot. Garden Ljubljana | 46.040.425 | 14.514.397 | s.n. (BGLjU) | MT732215 | MT732258 |
| I36 | *I. pallida* Lam. | Slovenia, Bot. Garden Ljubljana | 46.040.425 | 14.514.397 | s.n. (BGLjU) | MT732209 | MT732253 |
| I29 | *I. pseudacorus* L. | Croatia, Samobor | 45.812.279 | 15.824.715 | 2005/7 (BGZG) | MT732199 | MT732251 |
| I37 | *I. pseudacorus* L. | Slovenia, Bot. Garden Ljubljana | 46.040.425 | 14.514.397 | s.n. (BGLjU) | MT732200 | MT732250 |
| I28 | *I. pumila* L. | Hungary, Budaörs, Farkas Mt. | 47.274.252 | 18.573.042 | 2008/18 (BGZG) | MT732216 | MT732232 |
| I40 | *I.* *reichenbachii* Heuff. | Slovenia, Bot. Garden Ljubljana | 46.040.425 | 14.514.397 | s.n. (BGLjU) | MT732206 | MT732244 |
| I41 | *I.* x *rotschildii* Degen | Slovenia, Bot. Garden Ljubljana (from the l.c. Mt. Velebit, Croatia) | 46.040.425 | 14.514.397 | s.n. (BGLjU) | MT732211 | MT732257 |
| I32 | *I. sibirica* L. subsp. *erirrhiza* (Posp.) Wraber | Slovenia, Kavčiče, Bot. Garden Ljubljana | 46.040.425 | 14.514.397 | s.n. (BGLjU) | MT732204 | MT732249 |
| I34 | *I. sibirica*  subsp. *erirrhiza*  (Posp.) Wraber | Slovenia, Nanos Mt., Bot. Garden Ljubljana | 46.040.425 | 14.514.397 | s.n. (BGLjU) | MT732201 | MT732248 |
| I35 | *I. sibirica*  subsp. *erirrhiza*  (Posp.) Wraber | Slovenia, Alpski Vrt Juliana, Bot. Garden Ljubljana | 46.040.425 | 14.514.397 | s.n. (BGLjU) | MT732205 | MT732245 |
| I24 | *I. sibirica* L. subsp. *sibirica* | Croatia, Gubaševo | 46.001.820 | 15.534.910 | 2005/1 (BGZG) | MT732203 | MT732247 |
| I27 | *I. sibirica*  subsp. *sibirica* | Croatia, Slavonija, Petrijevci | 45.370.306 | 18.310.442 | 2005/2 (BGZG) | MT732202 | MT732246 |
