# Supplementary material for: Molecular Study of Selected Taxonomically Critical Taxa of the Genus Iris L. from the Broader Alpine-Dinaric Area
Source: Plants (Basel). 2020 Sep 18;9(9):1229. doi: 10.3390/plants9091229 (PMC7570032; doi:10.3390/plants9091229)
Supplement: Supplementary file 1 [file plants-09-01229-s001.zip › SUPPLEMENTARY FILES corr/Table S2.docx]

**Table S2.** Genotypes of the analysed *Iris* samples at eight microsatellite loci (allele sizes in bp).

| Sample/Locus | IM93 | | IM123 | | IM164 | | IM196 | | IM200 | | IM327 | | IM348 | | IM391 | | |
| --- | --- | --- | --- | --- | --- | --- | --- | --- | --- | --- | --- | --- | --- | --- | --- | --- | --- |
| I28 | 195 | 199 | 133 | 139 | 324 | 324 | 335 | 335 | 357 | 366 | 0 | 0 | 125 | 125 | 256 | 266 |  |
| I31 | 207 | 219 | 133 | 139 | 322 | 327 | 317 | 333 | 360 | 369 | 175 | 183 | 125 | 125 | 256 | 264 |  |
| I27 | 215 | 227 | 133 | 136 | 335 | 335 | 341 | 348 | 357 | 357 | 208 | 210 | 143 | 143 | 251 | 262 |  |
| I30 | 207 | 219 | 133 | 139 | 322 | 327 | 317 | 333 | 360 | 369 | 175 | 183 | 125 | 125 | 256 | 264 |  |
| I25 | 204 | 204 | 133 | 139 | 324 | 324 | 323 | 323 | 371 | 383 | 183 | 183 | 119 | 125 | 266 | 271 |  |
| I21 | 195 | 195 | 139 | 151 | 324 | 324 | 333 | 333 | 366 | 366 | n.a. | n.a. | 125 | 125 | 264 | 264 |  |
| I20 | 195 | 195 | 139 | 151 | 324 | 324 | 333 | 333 | 369 | 369 | n.a. | n.a. | 125 | 125 | n.a. | n.a. |  |
| I17 | 195 | 195 | 139 | 151 | 324 | 324 | 333 | 333 | 363 | 369 | 183 | 183 | 125 | 125 | 264 | 274 |  |
| I18 | 195 | 202 | 139 | 151 | 324 | 324 | n.a. | n.a. | 366 | 375 | 183 | 186 | 125 | 125 | n.a. | n.a. |  |
| I19 | 195 | 195 | 139 | 151 | 324 | 324 | 333 | 333 | 366 | 366 | n.a. | n.a. | 125 | 125 | n.a. | n.a. |  |
| I10 | 195 | 195 | 139 | 151 | 324 | 324 | 333 | 341 | 371 | 371 | 173 | 175 | 125 | 125 | 271 | 274 |  |
| I11 | 195 | 195 | 139 | 151 | 324 | 324 | 333 | 341 | 371 | 371 | 173 | 175 | 125 | 125 | 264 | 271 |  |
| I12 | 202 | 202 | 139 | 151 | 324 | 324 | 333 | 341 | 366 | 371 | 183 | 189 | 125 | 131 | 264 | 271 |  |
| I13 | 195 | 195 | 139 | 151 | 324 | 324 | 333 | 333 | 366 | 366 | 177 | 183 | 125 | 125 | 264 | 271 |  |
| I14 | 195 | 195 | 139 | 151 | 324 | 324 | 333 | 333 | 366 | 366 | 177 | 183 | 125 | 125 | 271 | 274 |  |
| I15 | 195 | 195 | 139 | 151 | 324 | 324 | 333 | 333 | 366 | 366 | 177 | 183 | 125 | 125 | 264 | 271 |  |
| I26 | 195 | 202 | 139 | 151 | 324 | 324 | 333 | 335 | 366 | 369 | 175 | 175 | 125 | 125 | 268 | 276 |  |
| I16 | 207 | 207 | 136 | 139 | 324 | 324 | 348 | 348 | 360 | 366 | 181 | 183 | 125 | 125 | 256 | 256 |  |
| I22 | 202 | 204 | 133 | 139 | 324 | 324 | 308 | 308 | 360 | 366 | 181 | 183 | 119 | 125 | 266 | 271 |  |
| I23 | 202 | 202 | 133 | 139 | 324 | 324 | 312 | 312 | 363 | 371 | 180 | 180 | 119 | 125 | 256 | 266 |  |
| I29 | 202 | 202 | 133 | 133 | 339 | 339 | 333 | 333 | 360 | 371 | 203 | 203 | 134 | 134 | 251 | 251 |  |
| I32 | 195 | 215 | 136 | 136 | 335 | 335 | 326 | 344 | 371 | 371 | 208 | 210 | 140 | 140 | 268 | 268 |  |
| I33 | 202 | 204 | 133 | 136 | 324 | 324 | 317 | 330 | 371 | 371 | 175 | 203 | 125 | 125 | 262 | 271 |  |
| I34 | 195 | 215 | 136 | 136 | 335 | 335 | 343 | 343 | 371 | 371 | 210 | 210 | 143 | 143 | 268 | 268 |  |
| I35 | 195 | 195 | 136 | 139 | 335 | 335 | 343 | 344 | 371 | 371 | 208 | 208 | 140 | 143 | 268 | 268 |  |
| I36 | 202 | 202 | 133 | 139 | 323 | 324 | 317 | 330 | 371 | 371 | 183 | 203 | 125 | 125 | 266 | 266 |  |
| I37 | 202 | 202 | 130 | 139 | 335 | 335 | 326 | 330 | 371 | 371 | 203 | 203 | 131 | 131 | 251 | 251 |  |
| I38 | 207 | 219 | 142 | 142 | 324 | 324 | 330 | 341 | 371 | 371 | 203 | 218 | 128 | 151 | 256 | 266 |  |
| I39 | 202 | 204 | 133 | 136 | 323 | 323 | 317 | 330 | 371 | 371 | 203 | 203 | 125 | 125 | 266 | 271 |  |
| I40 | 199 | 219 | 133 | 139 | 324 | 324 | 323 | 330 | 371 | 371 | 203 | 203 | 125 | 125 | 266 | 268 |  |
| I41 | 199 | 210 | 130 | 133 | 324 | 330 | 330 | 330 | 371 | 371 | 203 | 203 | 125 | 125 | 262 | 268 |  |

n.a. – not amplified
