# Supplementary material for: Molecular Study of Selected Taxonomically Critical Taxa of the Genus Iris L. from the Broader Alpine-Dinaric Area
Source: Plants (Basel). 2020 Sep 18;9(9):1229. doi: 10.3390/plants9091229 (PMC7570032; doi:10.3390/plants9091229)
Supplement: Supplementary file 1 [file plants-09-01229-s001.zip › SUPPLEMENTARY FILES corr/Table S3.docx]

**Table S3.** SSR and chloroplast markers used in the present molecular study of the Alpine-Dinaric taxa of the genus *Iris.*

| **SSR** | **Primer forward (5’-3’)** | **Primer reverse (5’-3’)** | **Rep. motif** | **Fluorescent**  **label** | **Ta (°C)** |
| --- | --- | --- | --- | --- | --- |
| IM93 | TTTGAGGACCAGATTGTACGG | ATGCCAACAAGGACATGAAAC | (ACC)_8_ | TAMRA | 58 |
| IM123 | GTTTCATGGAGGAGTTGCAGT | TATCTTGCGGGTCTTCTTGG | (TCT)_10_ | TAMRA | 58 |
| IM164 | ATATGTCAACAAACGCCAGGA | CTCGCATGTCAAGAACTCCAT | (AGC)_6_ | TAMRA | 53 |
| IM196 | GCATTCTTTGTGGAGAACCTG | CTGATCTCTTCCTCGGTCGT | (AAG)_7_ | FAM | 53 |
| IM200 | AAGCGAAATGGCGAATAAACT | AGATCAGAAGTCCGAGCACCT | (GAA)_23_ | HEX | 58 |
| IM327 | AAAGAGGAAGTGAGTTCTGGAGAG | GCTCCATTCTCAGGAGAGGTT | (GA)_12_ | HEX | 52 |
| IM348 | TCGAGTAGTCCCTCCCTCGT | GGAGAAGACGATGCTGAACC | (AGG)_7_ | FAM | 58 |
| IM391 | TTCTTCTTTATACCTTTCTTGTTTCC | TATTTGGCGACGATTGACTTC | (TTC)_12_ | TAMRA | 49 |
| **cP region** | **Primer name** | **Primer sequence (5’-3’)** |  | **Ta (°C)** |  |
| *ndhJ* | LP1-For | CATAGACCTTTRGGTTTYGA |  | 55 |  |
| *ndhJ* | LP4-Rev | ACCAATCCAASTATCRGGC |  | 55 |  |
| *rpoC1* | 1-For | GTGGATACACTTCTTGATAATGG |  | 55 |  |
| *rpoC1* | 3-Rev | TGAGAAAACATAAGTAAACGGGC |  | 55 |  |
